# Oral Cellular Homeostasis and Occupational Wellbeing in Healthcare Professionals Under the Lens of Salivary, Immune, and Microbiome Mechanisms

**DOI:** 10.3390/cells15050406

**Published:** 2026-02-26

**Authors:** Maria Antoniadou, Theodoros Varzakas

**Affiliations:** 1 Department of Dentistry, National and Kapodistrian University of Athens, 115 27 Athens, Greece; 2 Certified Systemic Analyst Program (CSAP), University of the Piraeus, 185 34 Piraeus, Greece; 3 Department of Food Science and Technology, University of the Peloponnese, Antikalamos, 241 00 Kalamata, Greece

**Keywords:** epithelial–immune crosstalk, mucosal homeostasis, biopsychosocial models, oxidative stress, occupational stress response, occupational wellbeing indices, salivary diagnostics in workplace medicine, oral homeostasis, salivary biomarkers, immune regulation, microbiome, healthcare professionals, resilience

## Abstract

**Highlights:**

- Positions oral cellular homeostasis as a biological interface linking occupational stress, immunity, and the oral microbiome.
- Integrates evidence from oral biology, nutrition, and occupational health.

**What are the main findings?**

- Occupational stress is associated with dysregulation of salivary biomarkers and oral immune–microbial balance.

**What are the implications of the main findings?**

- Oral biomarkers may support early detection of occupational strain.
- Integrated stress and diet-based interventions may enhance professional wellbeing.

**Abstract:**

Background: Healthcare professionals experience continuous biological and psychosocial stressors that may disturb oral and systemic homeostasis. Alterations in salivary secretion, mucosal immunity, and microbiome composition reflect adaptive cellular responses to chronic occupational stress. Understanding these mechanisms may provide a biological framework for resilience and wellbeing in everyday clinical practice. Objective: To narratively review the evidence linking oral cellular and molecular mechanisms—salivary biomarkers, epithelial and immune cell activity, and microbiome dynamics—with stress, fatigue, burnout, and wellbeing outcomes among healthcare professionals. Methods: This narrative review employed a PRISMA-guided literature search of PubMed, Scopus, Web of Science, and Cochrane Oral Health to enhance transparency and coverage across databases. Given the heterogeneity of study designs and outcomes, data were synthesized thematically without quantitative pooling or formal meta-analysis. Methodological strength was evaluated qualitatively, focusing on biomarker validity, sampling conditions, and conceptual relevance. Eligible designs included observational, experimental, and interventional studies. Results: Evidence from 99 studies suggests that chronic occupational stress elevates salivary cortisol, oxidative stress markers, and pro-inflammatory cytokines (IL-6, TNF-α), while reducing protective salivary immunoglobulin A and microbiome diversity. Balanced oral immune and microbial profiles were associated with better psychological adaptation and lower fatigue indices. Conclusions: Oral cellular homeostasis offers a promising window into the biological underpinnings of occupational stress and resilience in healthcare professionals. Systematic integration of salivary and mucosal biomarkers into workplace wellbeing programs could enhance early detection of dysregulated stress physiology. Future interdisciplinary research should bridge oral biology, occupational medicine, and mental health to strengthen sustainable wellbeing strategies across the health workforce.

## 1. Introduction

The oral cavity constitutes a highly dynamic mucosal barrier where epithelial cells, saliva, immune mediators, and a diverse microbial community interact continuously to maintain tissue homeostasis [1,2]. The structural and functional complexity of the oral mucosa reflects its composite defense architecture meaning physical, biochemical, microbiological, and immunological layers that collectively regulate pathogen exclusion and immune tolerance [3,4]. Recent advances in mucosal immunology indicate that epithelial–immune crosstalk influences not only local oral health but also systemic inflammatory and neuroendocrine dynamics, highlighting the integrative nature of oral barrier biology [5,6]. Within this ecosystem, the oral microbiome plays a central role, shaping immune maturation, barrier integrity, and inflammatory tone [7,8]. Disruption of microbial homeostasis has been linked to periodontal disease, mucosal inflammation, wound-healing dysregulation, and metabolic and neurocognitive outcomes, positioning oral microbial ecology as an important determinant of host systemic physiology [9,10]. Updated reviews have further shown that fluctuations in oral microbial composition interact with mucosal cytokine networks and epithelial signaling pathways, contributing to allostatic responses across the lifespan [11,12].

Complementing microbial mechanisms, saliva has emerged as a powerful, non-invasive biologic matrix that reflects both local oral status and systemic physiological states [13,14]. Salivary immunoglobulins, cytokines, proteomic signatures, and metabolites offer insight into mucosal immunity, inflammation, oxidative stress, and endocrine responses [15,16]. Μoreover, contemporary analyses highlight saliva’s diagnostic potential in infection, oncology, metabolic disorders, and mucosal immunopathology, reinforcing its value for personalized and longitudinal health monitoring [17,18]. Collectively, immunobiological evidence indicates that saliva integrates epithelial, microbial, and neuroendocrine signals, making it a sensitive biomarker matrix for stress-associated biological alterations [19,20].

Parallel to developments in oral biology, occupational wellbeing among healthcare professionals has become an urgent global concern, with high rates of burnout, chronic fatigue, circadian misalignment, depressive symptoms, and stress-related physiological dysregulation [21,22,23]. Systematic reviews consistently show that healthcare workers face elevated emotional and physical strain associated with workload pressure, moral distress, patient acuity, and disrupted work–rest cycles [24,25]. Importantly it is reported that biological studies increasingly demonstrate measurable physiological alterations, including immune suppression, cytokine activation, and neuroendocrine dysregulation, associated with occupational strain in healthcare environments [26,27]. Salivary biomarkers have been central in this research domain, with cortisol, Secretory Immunoglobulin A (SIgA), and pro-inflammatory cytokines widely used as indicators of occupational stress and allostatic load [28,29,30]. Methodological syntheses confirm that diurnal cortisol rhythms, cortisol awakening response, and stress-induced salivary cytokine fluctuations correlate strongly with occupational demands across nursing, emergency care, and shift-based medical work [31,32]. Evidence also suggests that mucosal immune components, including SIgA and epithelial-derived mediators, respond to acute and chronic stress exposure, further supporting the role of oral biomarkers in monitoring work-related physiological strain [14,33,34].

Despite these converging scientific trajectories, the interface between oral cellular homeostasis and occupational wellbeing in healthcare professionals remains substantially underexplored. Research in mucosal immunology has reported on the epithelial–immune–microbial signaling pathways but rarely considers psychosocial or occupational determinants as upstream modulators [1,5]. Conversely, occupational health studies employing salivary biomarkers often frame them as peripheral stress indicators rather than components of an interconnected mucosal system shaped by microbial and epithelial dynamics [28,30]. This disciplinary separation obscures potential bidirectional pathways through which occupational stress could influence oral homeostasis and through which oral mucosal biology might mediate resilience or vulnerability to chronic strain [33]. Further, healthcare professionals represent a uniquely relevant population in this context: continuous exposure to biological aerosols, pathogens, altered circadian patterns, and emotional labor may create distinct pressures on mucosal barrier immunity and microbiome stability [26,27]. Yet the extent to which occupational exposures reshape oral immune or microbial equilibrium, or how oral biomarkers might inform early detection of burnout and stress-related dysregulation, remains largely uncharacterized.

Accordingly, this narrative review aims to synthesize the current knowledge on oral cellular homeostasis, including salivary, immune, and microbiome pathways, and its potential links to occupational wellbeing in healthcare professionals. Specifically, we (i) summarize advances in oral mucosal immunity and microbiome science relevant to systemic regulation; (ii) review the diagnostic and physiological roles of salivary biomarkers in occupational contexts; and (iii) integrate emerging findings into a conceptual framework for understanding how oral biological mechanisms may interact with occupational stress, resilience, and wellbeing in healthcare settings.

Within this interdisciplinary context, the present review is guided by a central question: how oral cellular homeostasis, capturing salivary endocrine and immune biomarkers, epithelial barrier function, and oral microbiome dynamics, relates to occupational wellbeing, stress physiology, and burnout vulnerability in healthcare professionals? The PICO approach we used is in relation to healthcare professionals (P), exposure to oral cellular mechanisms, including salivary, immune, and microbiome pathways (I), that are examined in relation to occupational wellbeing outcomes such as stress physiology, burnout, and resilience (O), and compared with populations experiencing lower occupational strain where applicable (C).

We finally need to mention that rather than providing an exhaustive catalogue of oral biological pathways, this review adopts a selective and hypothesis-driven focus on salivary biomarkers, epithelial–immune signaling, and oral microbiome dynamics. These mechanisms are highlighted because they (i) are biologically sensitive to neuroendocrine stress signaling, (ii) operate at the interface between systemic physiology and local mucosal regulation, and (iii) can be repeatedly assessed in real-world occupational settings. Together, they form an integrated biological axis through which occupational stress may be biologically embedded and potentially modulated. This framework guides the synthesis that follows.

## 2. Materials and Methods

This work was designed as a narrative, theory-integrating review. Although PRISMA principles were followed to structure the search and selection process, the review does not aim to meet the full methodological requirements of a systematic review or meta-analysis [35]. The interdisciplinary scope of the research question, spanning oral biology, immunology, microbiome science, occupational health, and stress physiology, involves highly heterogeneous study designs, biomarkers, populations, and outcomes, rendering quantitative synthesis inappropriate [36]. Accordingly, no protocol registration was performed, no pooled effect-size estimation was attempted, and formal risk-of-bias tools were not uniformly applied across all study types. Instead, studies were appraised qualitatively, with emphasis on biological plausibility, methodological transparency, biomarker specificity, and relevance to the proposed conceptual framework.

### 2.1. Search Strategy and Information Sources

This review was designed as a selective, theory-driven narrative synthesis. Given the breadth and rapid expansion of the literature across oral biology, immunology, microbiome science, and occupational health, studies were prioritized based on their conceptual relevance to oral cellular homeostasis and stress biology, biological plausibility, and translational value, rather than exhaustive coverage of all available publications. The selection of databases was guided by the interdisciplinary nature of the research question, which covers all the above-mentioned topics. PubMed/MEDLINE was chosen as the primary biomedical database due to its comprehensive coverage of oral immunology, salivary diagnostics, stress physiology, and occupational health research, as well as its structured indexing through Medical Subject Headings (MeSH), which enabled precise mapping of core biological and psychosocial concepts. Scopus and Web of Science were included also to ensure broader interdisciplinary coverage and citation tracking, capturing relevant studies published in dental science, public health, psychology, and systems biology journals that may not be indexed in PubMed alone. Finally, Cochrane Oral Health was additionally searched to identify high-quality evidence syntheses and intervention-focused studies relevant to oral health and preventive strategies.

Search queries were tailored to the indexing systems and search functionalities of each database. In PubMed/MEDLINE, both MeSH and free-text keywords were employed to capture core concepts related to oral cellular homeostasis (including oral mucosa, oral immunity, saliva, and the oral microbiome), biological and stress-related biomarkers (such as cortisol, secretory IgA, cytokines, and metabolomics), and indicators of occupational wellbeing (including stress, burnout, fatigue, and healthcare professionals). Boolean operators (AND/OR) were applied to systematically combine conceptual domains. In Scopus and Web of Science, searches were conducted using keyword combinations applied to titles, abstracts, and author keywords in order to accommodate variations in terminology across disciplines. To ensure comprehensive coverage, the reference lists of all included articles and relevant narrative or systematic reviews were manually screened for additional eligible studies. Only peer-reviewed publications available in full English text were considered for inclusion. The full electronic search strategies for each database, including exact Boolean queries, date limits (January 2020–January 2026), language restrictions, and the final search date (31 January 2026), are provided in Supplementary Table S1.

### 2.2. Eligibility Criteria

Studies were eligible for inclusion if they met at least one of the following criteria: (1) examined oral epithelial, salivary, or microbiome mechanisms related to mucosal immunity or barrier homeostasis; (2) evaluated salivary biomarkers of stress, endocrine activity, immune activity, or inflammation; (3) assessed occupational stress, burnout, fatigue, psychological wellbeing, or physiological responses in healthcare professionals; (4) provided conceptual, mechanistic, or empirical insights bridging oral biology with systemic stress physiology. Overall, experimental, observational, interventional, and review studies were included. Exclusion criteria included non-oral biomarkers without relevance to mucosal immunity, non-healthcare populations unless offering transferable biological mechanisms, animal studies without translational value, non-peer-reviewed documents, conference abstracts, and publications not available in English.

### 2.3. Study Selection Process

Two reviewers independently screened titles and abstracts for relevance. Full texts were retrieved for all studies meeting or potentially meeting the inclusion criteria. Disagreements were resolved through discussion and consensus. Although narrative in design, the review followed PRISMA principles for transparency in search and selection [37]. The heterogeneity of study designs, biomarkers, and occupational outcomes precluded quantitative meta-analysis.

#### PRISMA Flow Description

**Figure 1:**
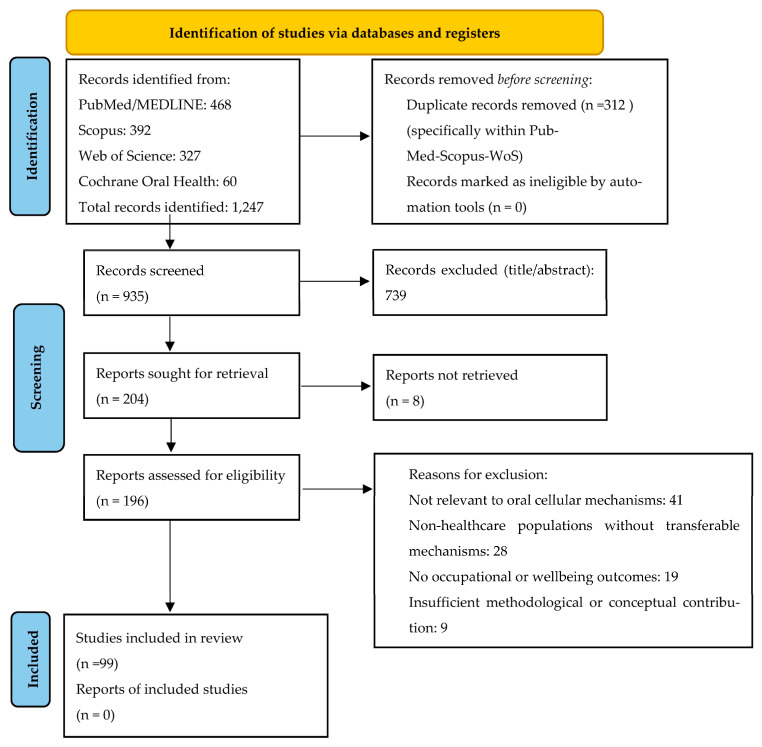
PRISMA flow chart of the study.

The literature search across PubMed/MEDLINE, Scopus, Web of Science, and Cochrane Oral Health initially yielded 1247 records dated from (January 2020–January 2026). After removal of 312 duplicate records, 935 unique articles remained for title and abstract screening. During the initial screening phase, 739 records were excluded because they did not address oral biology, salivary or mucosal mechanisms, occupational stress, or healthcare-related populations. The remaining 196 articles were assessed in full text for eligibility. Of these, 97 full-text articles were excluded for the following reasons: lack of relevance to oral cellular mechanisms (n = 41), focus on non-healthcare populations without transferable biological mechanisms (n = 28), absence of occupational or wellbeing-related outcomes (n = 19), or insufficient methodological or conceptual contribution (n = 9). A total of 99 studies finally met the inclusion criteria and were incorporated into the qualitative synthesis (Figure 1). These studies encompassed mechanistic immunology and microbiome research, salivary biomarker investigations, occupational health and wellbeing studies, and selected interventional trials relevant to stress biology and resilience in healthcare professionals. Given the heterogeneity of study designs, populations, biomarkers, and outcome measures, no quantitative meta-analysis was performed.

### 2.4. Data Extraction and Thematic Organization

Data extraction focused on: (i) study design; (ii) population characteristics; (iii) oral biomarkers assessed (salivary cortisol, SIgA, cytokines, microbiome profiles, epithelial–immune markers); (iv) occupational or psychological outcomes (burnout, stress, fatigue, circadian disruption); and (v) mechanistic insights linking oral biology to systemic physiology. Thematic synthesis was applied, grouping emerging evidence into three major domains: (1) oral mucosal immunity and epithelial barrier regulation; (2) oral microbiome composition and its interaction with immunological and neuroendocrine pathways; (3) salivary biomarkers as indicators of stress physiology and occupational load in healthcare professionals.

**Figure 2:**
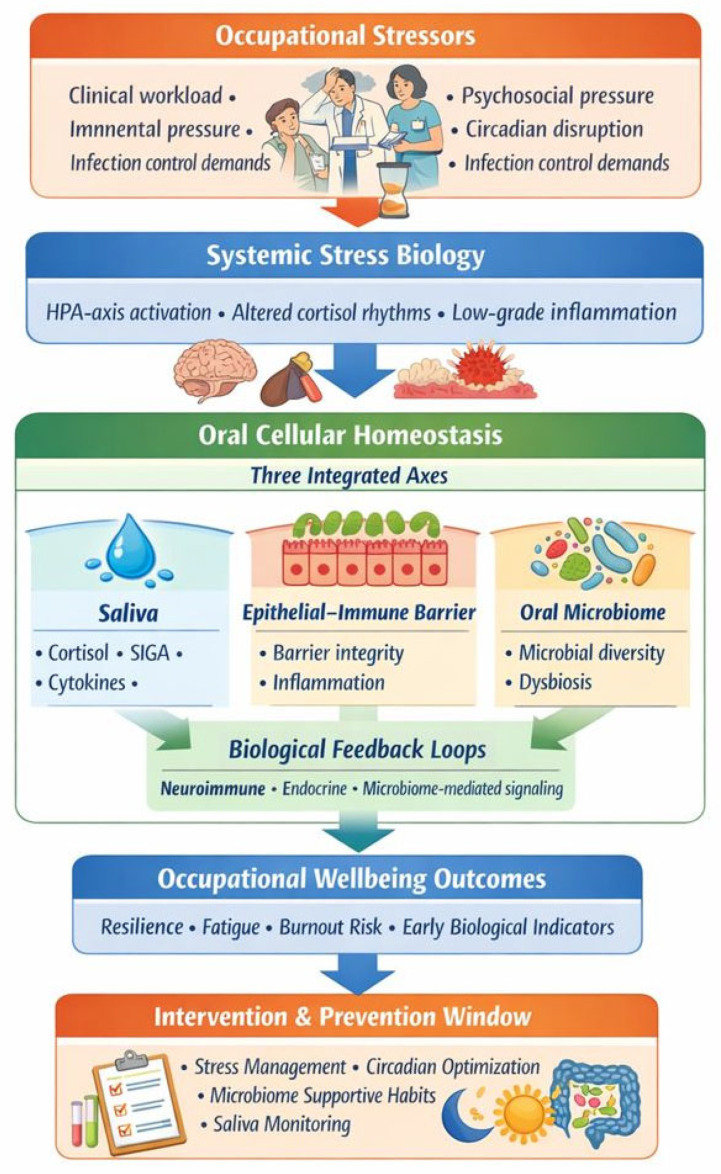
Conceptual overview of how occupational stress engages systemic stress biology and interacts bidirectionally with oral salivary, epithelial–immune, and microbiome processes. The schematic illustrates a simplified and integrative model highlighting potential, context-dependent interactions rather than linear or deterministic pathways. Alterations in oral cellular homeostasis may reflect and potentially influence occupational wellbeing, positioning the oral cavity as an accessible biological interface for hypothesis generation, longitudinal monitoring, and prevention-oriented research in healthcare professionals (This figure: created with BioRender.com; accessed on 5 December 2025).

Cross-domain integration was then performed to construct a conceptual model describing potential bidirectional pathways between oral cellular homeostasis and occupational wellbeing. Τhe model derived out of this search can be seen in Figure 2.

### 2.5. Quality Considerations and Methodological Limitations

Given the narrative design of this review and the substantial heterogeneity of the included literature, the uniform application of formal risk-of-bias assessment tools (such as ROBINS-I, the Newcastle-Ottawa Scale, or RoB 2) was not considered appropriate across all study types. Instead, methodological quality was evaluated qualitatively, with attention to key features relevant to the research question, including sample size adequacy, validity and specificity of oral and salivary biomarkers, conditions and timing of saliva collection, analytical and laboratory techniques, control for diurnal and contextual variation, and the operationalization and measurement of occupational stressors. Particular consideration was given to methodological transparency and consistency in defining biological and psychosocial outcomes. Variability in biomarker assays, microbiome sequencing platforms, bioinformatic pipelines, and psychological assessment instruments was recognized as a major source of methodological heterogeneity, limiting direct comparability across studies [38]. These methodological differences were explicitly taken into account during the synthesis process and informed the relative weighting and interpretation of findings within and across thematic domains. Rather than serving as grounds for exclusion or quantitative weighting, methodological limitations were treated as contextual factors informing interpretation, consistent with narrative synthesis approaches.

### 2.6. Review Scope and Conceptual Orientation

This review does not aim to present an exhaustive systematic catalogue of all relevant studies; rather, it integrates high-quality and mechanistically informative evidence to outline how oral immune–microbial–endocrine dynamics may intersect with occupational stress biology. The approach reflects the inherently interdisciplinary nature of the topic and seeks to advance conceptual understanding, generate hypotheses, and identify gaps requiring targeted empirical investigation.

## 3. Results

Across the final body of evidence included in this review, studies were organized into six major thematic domains reflecting the core components of oral cellular homeostasis and their intersections with occupational wellbeing: (i) oral mucosal immunity and epithelial barrier regulation, (ii) oral microbiome composition and its systemic physiological links, (iii) integrated evidence on oral microbiome, mucosal immunity, saliva, and systemic-stress biology, (iv) occupational stress, wellbeing & biomarkers, (v) dietary, nutritional, and food-related modulators of oral cellular homeostasis, and (vi) interventions targeting stress biology, oral homeostasis, and occupational wellbeing. This thematic categorization allowed for the synthesis of highly heterogeneous research designs, ranging from mechanistic immunology and microbiome analyses to cross-sectional wellbeing studies and randomized controlled interventions, while preserving the mechanistic coherence necessary for interpreting how oral biological pathways may mirror or modulate occupational stress physiology. Table 1 presents the key articles mapped to each domain, summarizing study characteristics, methodological approaches, and principal findings relevant to the conceptual model of oral homeostasis as a potential biomarker and resilience interface in healthcare professionals.

### 3.1. Oral Mucosal Immunity and Epithelial Barrier Responses Under Stress

Across the selected literature, oral mucosal immunity emerges as a tightly regulated, multilayered system, where epithelial integrity, innate immune surveillance, and resident microbiota operate as an integrated functional unity [34]. Mechanistic analyses demonstrate that homeostasis depends on continuous bidirectional communication between epithelial cells, commensal microorganisms, and immune mediators, a process mediated by cytokines, antimicrobial peptides, and pattern-recognition receptors that calibrate tolerance and inflammation in real time [1]. The oral mucosa is described not merely as a physical barrier but as a metabolically active immune organ whose structural and immunological layers respond rapidly to environmental disturbances, microbial pressures, and endocrine changes [15,34,46,48].

Detailed immunological mapping further supports that the oral barrier consists of an epithelial–microbial–immune triad capable of immediate adaptation via tight-junction remodeling, mucin secretion, and activation of innate effectors such as defensins and secretory IgA [3,39]. Comparative mucosal analyses reveal that, unlike the gastrointestinal tract, the oral mucosa is exposed to higher microbial diversity, rapid antigenic turnover, and continuous mechanical and chemical stress, necessitating heightened innate immune responsiveness and faster epithelial renewal [5]. Central to this defense are specialized lymphocyte subsets, most notably γδ T cells, which contribute to epithelial repair, microbial containment, and immune tolerance, and which are sensitive to neuroendocrine modulators and stress-related dysregulation [6]. Parallel advances in neuroimmune biology show that sensory innervation of the oral mucosa is intimately connected with immune activation: neuropeptides, sympathetic mediators, and glucocorticoids influence epithelial cytokine expression, dendritic cell priming, and the balance between pro- and anti-inflammatory responses [7]. Under conditions of sustained stress, these neuroimmune pathways can induce transient barrier weakening, alter antimicrobial output, and shift inflammatory thresholds [1,7]. Further immunological work maps how epithelial cells act as both structural and immune sentinels, initiating rapid signaling through Toll-like receptors (TLRs), inflammasome components, and chemokine networks that guide leukocyte recruitment and microbial containment [34]. When epithelial and immune homeostasis is perturbed, whether through microbial imbalance, endocrine fluctuation, or chronic systemic stress, the mucosa demonstrates measurable shifts in permeability, cytokine gradients, oxidative stress, and immune-cell composition [1,3]. These findings indicate overall that the oral mucosa is a dynamic neuroimmune barrier whose cellular homeostasis is exquisitely sensitive to systemic physiological stressors, including those commonly observed in healthcare work environments. This establishes a strong mechanistic basis for considering epithelial–immune–microbial interactions as potential early biological indicators of occupational strain and resilience [39].

**Figure 3:**
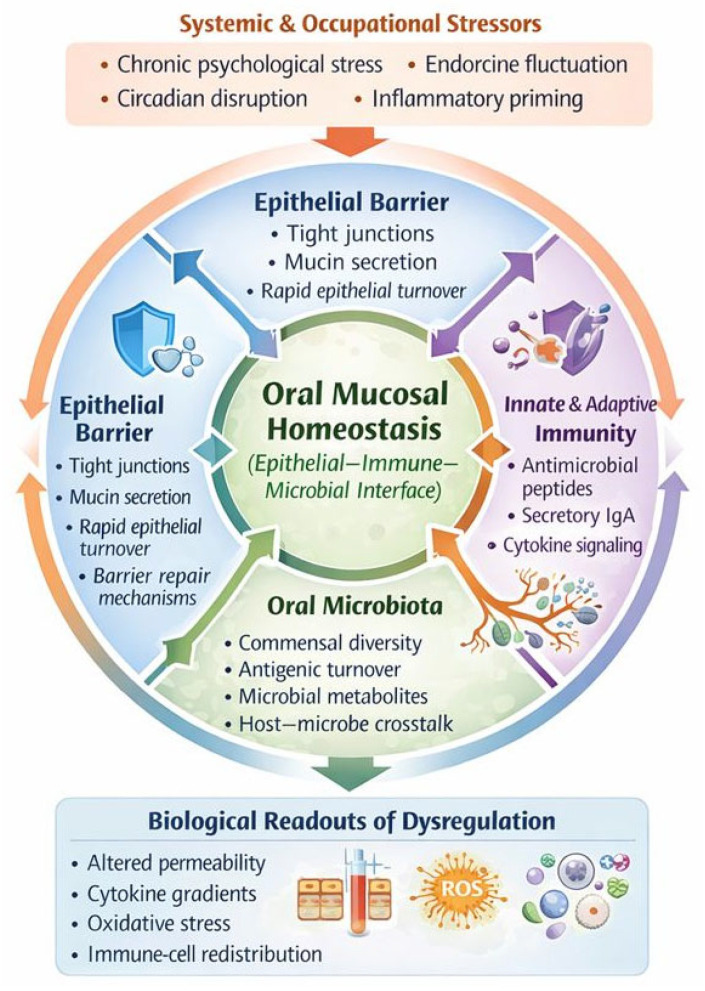
Conceptual model of oral mucosal immunity as a neuroimmune barrier system integrating epithelial, immune, and microbial components. The schematic depicts a simplified and integrative framework illustrating potential, context-dependent interactions among systemic and occupational stressors, epithelial barrier function, innate and adaptive immune responses, and oral microbiota dynamics. These interactions are shown as bidirectional and adaptive rather than linear or deterministic, with dysregulation at any level potentially reshaping oral cellular homeostasis and downstream biological readouts. The model is intended to support conceptual integration and hypothesis generation, while acknowledging substantial biological variability, temporal dynamics, and uncertainty across individuals and occupational contexts (This figure: created with BioRender.com; accessed on 5 December 2025).

As summarized in Figure 3, these findings converge on a model in which the oral mucosa can be conceptualized as a dynamic epithelial–immune–microbial interface regulated by continuous bidirectional signaling among epithelial cells, innate and adaptive immune mechanisms, resident microbiota, and neuroendocrine inputs. Systemic and occupational stressors modulate these interactions through endocrine and neural pathways, and are associated with measurable changes in barrier integrity, immune signaling, and inflammatory balance [7,11,12].

The above-mentioned model illustrates the mechanistic basis by which oral cellular homeostasis may act as a sensitive biological interface for stress-related dysregulation. However, it is important to emphasize that, despite strong mechanistic plausibility, these epithelial–immune pathways have not yet been clinically validated as intervention targets in occupational or healthcare settings, and their modulation under real-world conditions remains largely unexplored.

### 3.2. Oral Microbiome Composition and Oral–Systemic Biological Interactions

Across the contemporary literature, oral cellular homeostasis emerges as the product of tightly interconnected microbial–host interactions operating at the mucosal surface and respond sensitively to systemic, hormonal, environmental, and psychosocial influences [28,33]. Conceptual immunology frameworks describe the mucosal surface as an adaptive immune ecosystem in which commensal microbiota actively shape immune maturation, tolerance, and inflammatory thresholds from early life to aging, rather than acting as passive colonizers [7,14]. This perspective is reinforced by multi-omics and longitudinal evidence linking oral health trajectories with cognitive aging and social determinants, suggesting that oral biological integrity reflects cumulative biological and environmental exposures over time [9,10]. Within this framework, periodontitis-related research provides a concrete disease model illustrating how microbial imbalance, immune dysregulation, and systemic signaling intersect [8]. Narrative and biomarker-focused reviews consistently demonstrate that alterations in the oral microbiome are mirrored by shifts in salivary inflammatory and metabolic markers, supporting saliva as a functional readout of ongoing host–microbe conflict [8,60]. Importantly, causal inference approaches strengthen this association further: Mendelian randomization analyses suggest that gut microbiota composition may exert a direct causal influence on periodontitis risk, highlighting bidirectional oral-gut microbial crosstalk rather than isolated local pathology [40]. Parallel systematic analyses of endodontic and prosthodontic conditions further show that clinical interventions and anatomical changes, such as root canal infection or denture use, reshape microbial ecology toward more inflammation-prone configurations, emphasizing the sensitivity of oral ecosystems to both biological and mechanical stressors [9,42,43].

In particular, hormonal and immune signaling represent critical modulatory layers within this system. Clinical and experimental syntheses demonstrate that endocrine environments, particularly stress- and sex-hormone fluctuations, reshape oral microbial composition and immune tone, providing a mechanistic bridge between psychosocial stress, HPA-axis activity, and oral dysbiosis [11,17]. At the cellular level, as detailed in Section 3.1, epithelial cells are increasingly recognized as active immune sentinels that integrate microbial signals, cytokine gradients, and neuroendocrine inputs to regulate barrier integrity and inflammatory responses [34]. Transcriptomic and proteomic studies of saliva extend this view, showing that salivary RNA and protein profiles capture both local immune activity and systemic physiological states, effectively positioning saliva as a multi-layered biosensor rather than a passive secretion within the context of host–microbiome interactions [18,20]. The functional relevance of these interactions becomes particularly evident in the context of immune challenge and environmental stress. Studies of viral infection, vaccination, and inflammatory disease demonstrate that oral mucosal immunity, in concert with microbial ecology, plays a decisive role in shaping systemic immune responses, viral susceptibility, and inflammatory resolution, with saliva-mediated mechanisms contributing to immune defense and pathogen control [46,47,48]. Microbiome-derived metabolites, including indole derivatives and short-chain fatty acids, further modulate immune signaling and inflammatory balance, reinforcing the concept that microbial metabolism constitutes a key regulatory layer of host immunity [50].

Finally, emerging interventional and environmental studies highlight the plasticity of oral biological systems. Nutritional interventions, micronutrient supplementation, and enriched environments have been shown to modulate salivary cortisol, immune markers, microbiome composition, and even emotional regulation, illustrating that oral cellular homeostasis responds measurably to modifiable behavioral and environmental inputs [45,51,54]. In sum, these findings converge on a unified model in which microbiome-driven processes are integrated within saliva as a composite biological matrix, reflecting context-dependent microbial, immune, and neuroendocrine signals at the oral–systemic interface and supporting oral biology as a mechanistic hub for stress-related physiological adaptation rather than a direct translational target.

### 3.3. Salivary Biomarkers of Endocrine, Immune, and Metabolic Activity

The evidence base on saliva converges on a central premise: saliva functions as an integrative biological matrix that reflects the convergence of endocrine, immune, and microbial processes occurring at the oral–systemic interface and therefore serves primarily as a readout, and secondarily as a modulator, of mucosal homeostasis [13,39]. Narrative syntheses emphasize that salivary composition captures both local oral processes and systemic physiological states, supporting its use in repeated, low-burden monitoring contexts [13,16,17,18]. Mechanistically, saliva contains a layered repertoire of immune mediators (e.g., SIgA, cytokines, antimicrobial peptides), endocrine markers (e.g., cortisol), and molecular cargo (metabolites, proteins, nucleic acids, and extracellular vesicles), enabling multi-scale profiling of stress biology, immune tone, and disease susceptibility [14,17,18,40]. Importantly, methodological work suggests that salivary biomarker interpretability depends on sampling conditions, diurnal timing, assay characteristics, and contextual exposures, which are critical when translating saliva-based measures into occupational health monitoring [16,30].

From an immunological standpoint, saliva contributes to frontline mucosal immune defense: natural and inducible responses in the oral cavity rely on sIgA, cytokine signaling, and innate antimicrobial factors that collectively regulate microbial colonization and support epithelial integrity under continuous antigenic pressure [14,84]. Proteomic evidence further indicates that salivary proteins participate directly in host–microbe regulation by modulating adhesion, biofilm dynamics, and pathogen neutralization, linking molecular composition to ecological stability [18,49]. This ecological role is reinforced by microbiology-focused reviews showing that saliva is not merely a passive medium but contributes to stabilization of the oral microbiome through nutrient provisioning and selective antimicrobial pressure, thereby shaping community resilience and inflammatory thresholds [39,49,50]. In parallel, salivary metabolomics expands the diagnostic scope beyond single analytes toward systems-level signatures: metabolomic patterns reflect inflammatory and metabolic states bridging oral and systemic disease processes, while also offering potential for risk stratification and monitoring. Transcriptomic approaches similarly position the salivary transcriptome as an accessible window into local and systemic gene expression programs relevant to immune activation and stress responses [55].

Emerging salivary “next-generation” biomarkers strengthen this integrative model. Reviews of salivary exosomes highlight their role as diagnostic and signaling mediators in oral disease, expanding saliva’s utility from biochemical readouts to intercellular communication platforms [70]. At the endocrine level, saliva-based hormone diagnostics consolidate the rationale for cortisol monitoring in stress-related contexts, supporting the linkage between HPA-axis dynamics and mucosal immune modulation [17,30]. Collectively, these lines of evidence support the interpretation that salivary immune–endocrine–omic profiles can capture early biological dysregulation and recovery dynamics, potentially complementing subjective measures and enabling translational monitoring frameworks in high-demand healthcare environments [13,16,39,105]. Further, diagnostic reviews centered on hormone detection highlight saliva as a matrix for measuring free (biologically active) fractions of steroid hormones, particularly cortisol, with good correlation to serum levels and strong practical advantages for field and occupational studies [17]. Landmark work in occupational and environmental medicine established salivary cortisol as a core indicator of HPA-axis activation and stress-related physiological burden [28]. More recent methodological syntheses refined this picture, emphasizing that diurnal patterns, the cortisol awakening response, sampling timing, and assay characteristics critically influence interpretability, and must therefore be carefully standardized in research linking occupational conditions to salivary readouts [30,31]. Together, these findings position salivary cortisol and related hormones as key tools for quantifying the endocrine dimension of occupational stress biology.

Metabolomic and transcriptomic studies extend the scope of salivary biomarkers beyond individual molecules toward system-level signatures. Metabolomics work demonstrates that salivary metabolic profiles track systemic metabolic states, inflammation, and disease risk, offering a multiplex, hypothesis-generating view of physiological perturbations [15]. Parallel transcriptomic approaches indicate that salivary RNA expression patterns mirror both local and systemic gene regulation. These signatures include stress- and immune-related pathways, supporting saliva as a minimally invasive window into integrated biological responses [46,48,55,56,57,58,59]. These high-dimensional approaches are particularly relevant to occupational settings, where chronic low-grade stress may produce subtle, multi-parameter shifts rather than large changes in any single biomarker [18,58,62].

Importantly, accumulating evidence indicates that the interpretive value of salivary biomarkers depends not only on analyte selection, but also on sampling timing, methodology, and population characteristics [59,60]. Narrative syntheses mention the impact of collection methods, circadian timing, flow rate, stimulation, storage, and analytical platform on biomarker reliability, stressing the need for strong protocol design in both research and applied monitoring [13,63,64,65,66]. Trials that manipulate environmental or behavioral conditions illustrate this point concretely: interventions such as tele-yoga, lifestyle modification for night-shift workers, or enriched environments have demonstrated measurable changes in salivary cortisol, inflammatory markers, and microbiota-related parameters, linking modifiable workplace or lifestyle factors to salivary biomarker trajectories [26,27,32]. These findings reinforce saliva’s responsiveness to psychosocial and occupational exposures [78,79,80,81,82,83]. However, these findings primarily demonstrate biomarker responsiveness rather than validated clinical efficacy, reporting on the need for controlled longitudinal studies.

**Figure 4:**
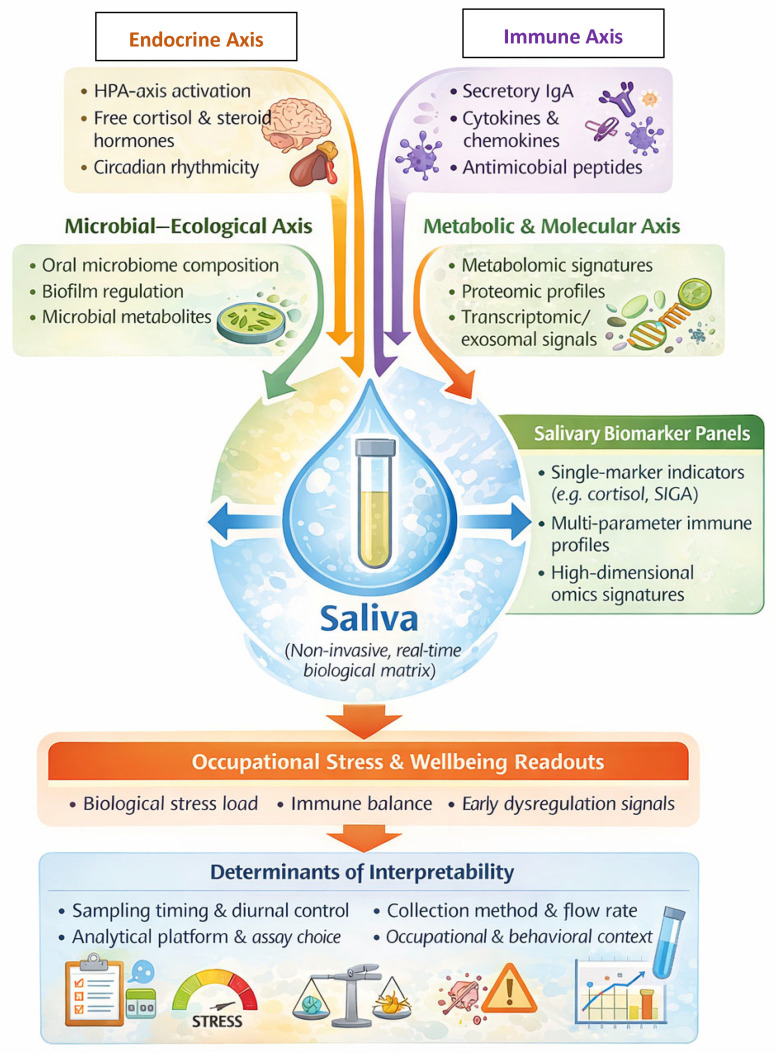
Conceptual representation of saliva as an integrative biosensor reflecting interactions among endocrine, immune, microbial-ecological, and metabolic-molecular axes. The schematic illustrates how salivary biomarker panels may capture context-dependent, multidimensional biological signals associated with occupational stress and wellbeing, rather than linear or deterministic pathways. Arrows denote bidirectional and convergent information flow from multiple physiological systems into saliva as a non-invasive biological matrix. The figure emphasizes that interpretability of salivary readouts is influenced by sampling conditions, analytical choices, and occupational context, and that such readouts are intended to support hypothesis generation, longitudinal monitoring, and prevention-oriented research rather than definitive diagnostic or predictive use (This figure: created with BioRender.com; accessed on 10 December 2025).

Taken together, the available evidence portrays saliva as a multi-layered biosensor that captures endocrine activation, immune balance, microbial interaction, and metabolic status within a single, non-invasive biological matrix [39]. Hormonal dynamics, mucosal immune activity, microbiome-derived signals, and high-dimensional molecular profiles converge to produce measurable salivary biomarker patterns. The interpretability of these patterns depends on sampling conditions, circadian timing, and analytical methodology. This framework highlights saliva’s value for capturing the biological imprint of occupational stress and for exploring physiological responses to workplace and lifestyle interventions (Figure 4).

For the purposes of occupational wellbeing in healthcare professionals, the above suggests that carefully selected and standardized salivary panels, combining cortisol dynamics, SIgA, cytokine profiles, and possibly metabolomic or transcriptomic signatures, could provide a sensitive window into the biological imprint of chronic work-related stress, as well as into the efficacy of resilience-building interventions [76,77,78]. Accordingly, salivary biomarkers should be interpreted as dynamic, context-dependent readouts of integrated stress biology, rather than as isolated or diagnostic indicators.

### 3.4. Occupational Wellbeing in Healthcare and Its Intersection with Oral Biology

The literature on occupational wellbeing consistently portrays healthcare environments as high-strain systems in which chronic psychological load, organizational pressures, and moral distress accumulate and are associated with measurable biological dysregulation [22,41,85,86]. Scoping and systematic reviews document high rates of burnout, depression, anxiety, and suicidal ideation among healthcare professionals, with nurses, physicians, and other frontline staff showing persistent vulnerability across settings and countries [22,87,88,89,90,91,92,93,94,95]. Beyond psychological outcomes, these reviews emphasize that occupational distress is biologically embodied through alterations in circadian rhythms, hypothalamic–pituitary–adrenal (HPA)-axis activity, immune function, and systemic inflammation, placing healthcare workers at long-term risk for cardiometabolic and neuropsychiatric morbidity [22].

Within this broader context, emerging work in dentistry and academic health settings provides more fine-grained insight into how stress is experienced and modulated. Cross-sectional data from Greek dentistry and nursing students indicate substantial burdens of stress, anxiety, and depression two years after the COVID-19 pandemic, but also highlight resilience, hope, and spiritual wellbeing as potential protective resources that may buffer the impact of academic responsibilities [85]. Similarly, comparative work on academic dental and nursing personnel shows that indices of quality of life and wellbeing are closely related to perceived quality of services and academic functioning, suggesting that staff wellbeing is not a “soft” outcome but interwoven with educational performance and service delivery [86,104,105]. Together, such studies position oral health professionals and related academic staff as a meaningful subgroup within the broader healthcare workforce, facing both typical healthcare stressors and profession-specific demands (clinical precision, infection control, patient-facing communication, and teaching responsibilities).

Interventional and integrative studies further delineate the modifiable components of occupational stress biology. An integrative review of non-pharmacological interventions for healthcare workers reports that structured stress-reduction strategies, such as mindfulness, relaxation practices, and behavioral programs, can yield improvements not only in self-reported wellbeing but also in physiological markers of stress and immune function [25]. Furthermore, a pilot randomized controlled trial on tele-yoga for healthcare workers on COVID-19 duty demonstrated reductions in burnout and improvements in mental health indices, accompanied by favorable shifts in immune markers, underlining the feasibility of remote, biologically relevant interventions within experimental and pilot settings [26]. Similarly, a randomized crossover lifestyle intervention in female night-shift healthcare workers reported benefits in metabolic and mental health parameters, with concomitant changes in salivary cortisol and related biomarkers, directly linking occupational scheduling, behavioral modification, and measurable stress physiology [27]. Such findings collectively indicate that occupational distress is not a fixed trait but a modifiable state, with interventions capable of recalibrating both psychological and biological endpoints.

Salivary and mucosal biomarkers sit at the intersection of these phenomena. Landmark work on salivary biomarkers in occupational and environmental medicine positioned saliva, particularly salivary cortisol, as a widely used, central non-invasive tool for assessing acute and chronic stress load in workplace settings [28]. More recent methodological syntheses refine this paradigm by articulating how sampling protocols, diurnal timing, and assay characteristics critically shape the interpretation of salivary cortisol and related markers in biobehavioral research [30]. The cortisol awakening response (CAR), in particular, has been identified as a sensitive, relatively stable index of HPA-axis activation under chronic stress, further reinforcing saliva’s utility for tracking occupational strain [31]. Randomized trials manipulating environmental richness in early life provide additional proof-of-principle that contextual factors can alter both salivary cortisol profiles and microbiota-related parameters, illustrating the plasticity of stress-related biomarkers in response to environmental modulation [32].

When viewed alongside the evidence on oral mucosal immunity (Section 3.1), microbiome dynamics (Section 3.2), and the complex biomarker landscape of saliva (Section 3.3), these occupational studies suggest a coherent, though still underdeveloped, picture. Healthcare workers, including dental professionals and academic staff-experience chronic psychosocial stress that is biologically inscribed through endocrine, immune, and metabolic pathways, many of which can be accessed via oral biomarkers [22,96,97]. At present, most occupational studies treat saliva primarily as a convenient stress readout [87,98,99]. However, the underlying science of oral epithelial barriers, immune responses, and microbial ecosystems indicates that the oral cavity itself may contribute to processes of stress adaptation and vulnerability [100]. This creates a conceptual bridge between occupational wellbeing and oral cellular homeostasis, supporting the hypothesis that dentists and other healthcare professionals could be monitored, and potentially better characterized, through integrated panels of salivary, immune, and microbiome indicators that capture both psychological load and mucosal resilience over time. Nevertheless, the current data primarily demonstrate proof-of-concept effects, and their scalability, durability, and relevance to oral cellular homeostasis remain to be established.

### 3.5. Dietary, Nutritional, and Food-Related Modulators of Oral Cellular Homeostasis

Dietary and nutritional factors constitute important upstream modulators of oral cellular homeostasis, acting through interconnected immune, microbial, and metabolic pathways that parallel mechanisms described in intestinal and systemic biology [88,89]. Systematic and narrative syntheses demonstrate that diet shapes oral epithelial integrity, mucosal immune tone, and microbial ecology by regulating substrate availability, inflammatory signaling cascades, and redox balance within the oral environment [88,89,92]. Diets enriched in fermentable fibers, polyphenols, and bioactive micronutrients favor the production of microbial-derived metabolites, most notably short-chain fatty acids (SCFAs), which are associated with anti-inflammatory signaling, barrier support, and immunoregulatory effects, whereas highly processed, sugar-rich dietary patterns are consistently associated with dysbiosis, oxidative stress, and pro-inflammatory signaling in oral tissues [89,90,91,92,93]. Emerging evidence further suggests that dietary inflammatory potential is associated with distinct oral and gut microbiota profiles and systemic inflammatory markers, supporting the concept that diet-driven inflammation influences host–microbiome interactions across mucosal sites [90]. Mechanistic analyses highlight SCFAs as key mediators linking dietary substrates to epithelial energy metabolism, immune modulation, and microbial stability in both the oral cavity and the gastrointestinal tract [91,93].

Within this framework, prebiotics, probiotics, postbiotics, and synbiotics represent complementary nutritional strategies capable of reshaping microbial composition, strengthening epithelial defense mechanisms, and calibrating innate and adaptive immune responses [88,89]. Although much of the mechanistic evidence originates from gastrointestinal models, converging data suggest that analogous pathways may operate in the oral cavity, where constant microbial exposure and rapid epithelial turnover render oral tissues particularly responsive to dietary inputs [91,92,93]. Collectively, these findings position diet not merely as a background lifestyle factor but as a biologically relevant modulator of oral cellular homeostasis, with implications for inflammation control, immune balance, and stress-related vulnerability in healthcare populations [94]. Building on these diet–microbiome–immune interactions, postbiotics have emerged as a promising class of bioactive compounds through which nutritional modulation may exert more targeted effects on oral cellular homeostasis.

#### 3.5.1. Postbiotics

Postbiotics are defined as preparations of non-viable microbial cells, cell components, or metabolites that confer health benefits to the host, comprising bioactive compounds such as short-chain fatty acids (SCFAs), exopolysaccharides (EPS), bacteriocins, antioxidant enzymes, surface layer proteins, and bacterial lysates. They interact with host pattern-recognition receptors and epigenetic pathways, thereby supporting epithelial barrier function, regulating innate and adaptive immune responses, and modulating host gene expression [106]. Their health-promoting potential has been explored in the context of metabolic regulation, immune modulation, and mental health, while also addressing limitations associated with microbial viability and antibiotic resistance transfer [107,108].

A growing body of evidence indicates that postbiotics exert antioxidant, anti-inflammatory, immunomodulatory, and metabolic effects that are relevant to both systemic and oral health, supporting their role as biologically active mediators of diet–microbiome–host interactions [95,96,109,110]. Key postbiotic components, including short-chain fatty acids (SCFAs), polysaccharides, proteins, and bacterial cell wall fragments, contribute to microbial balance and protection against immune-mediated and inflammatory disorders by modulating epithelial function and immune signaling pathways [97,111,112]. In particular, *Lactobacillus*-derived postbiotics have demonstrated promising effects on glucose regulation, obesity-related metabolic dysregulation, and intestinal homeostasis, highlighting their relevance in the context of metabolic–immune crosstalk [98,113,114]. Complementary to postbiotics, prebiotics-defined as non-digestible substrates such as inulin and oligofructose-selectively stimulate beneficial microbial taxa, enhance SCFA production, and promote metabolically favorable shifts in bile acid and fatty acid metabolism, thereby reducing the risk of metabolic and inflammatory disorders [99,115,116]. Furthermore, synbiotics, which combine probiotics and prebiotics, optimize microbial survival and functionality, yielding synergistic effects on microbial stability and host immune responses [99]. Conclusively, the integrated use of prebiotics, probiotics, postbiotics, and synbiotics represents a complementary and biologically coherent strategy for modulating the microbiota and its downstream immune and metabolic effects, with emerging relevance for oral cellular homeostasis and disease prevention, although further mechanistic and long-term clinical studies are warranted [117,118].

Moreover, exopolysaccharides (EPSs) are high-molecular weight carbohydrate polymers produced by a range of commensal and probiotic microorganisms and are increasingly recognized as bioactive mediators of host–microbe interactions across mucosal surfaces [119]. Accumulating evidence demonstrates that EPSs exert a broad spectrum of health-promoting effects, including inhibition of pathogenic adhesion to epithelial surfaces, reinforcement of mucosal barrier integrity, and modulation of innate and adaptive immune responses [120,121,122]. Structurally, EPSs are classified into homopolysaccharides (HoPSs), composed of repeating units of a single monosaccharide, and heteropolysaccharides (HePSs), consisting of complex repeating units of different monosaccharides; this structural diversity is closely linked to functional specificity in epithelial interaction and immune modulation [123,124]. Recent mechanistic studies indicate that EPSs can interact directly with epithelial cells to enhance tight-junction protein expression and mucin production, thereby strengthening barrier function and contributing to mucosal homeostasis [122,125]. In parallel, EPSs have been shown to modulate immune signaling pathways by attenuating pro-inflammatory cytokine production and promoting regulatory immune responses, including effects on dendritic cell activation and T-cell polarization [126,127]. Beyond immunoregulation, experimental studies have reported antiproliferative and antioxidant effects of certain EPSs, primarily in preclinical models, with Lactobacillus-derived EPSs receiving particular attention due to their favorable safety profile [128,129,130]. Collectively, contemporary evidence supports EPSs as multifunctional microbial metabolites that contribute to mucosal barrier stability, immune balance, and epithelial resilience, with emerging relevance for oral cellular homeostasis within diet–microbiome–host interaction frameworks.

Short-chain fatty acids (SCFAs) are absorbed and transported via the bloodstream to peripheral tissues, where they exert systemic effects and act as key mediators linking the microbiota to host immune and metabolic regulation [131,132]. In intestinal epithelial cells, SCFAs contribute to energy metabolism through acetyl-CoA production and support immune cell function [133,134].In intestinal epithelial cells (IECs), acetyl-CoA is the metabolic product of SCFAs via the TCA cycle, offering energy production and immune cell function [133,134].

Additionally, bacteriocins are ribosomally synthesized antimicrobial peptides produced by Gram-positive and Gram-negative bacteria, exhibiting narrow or broad inhibitory spectra against competing microorganisms [135]. Their primary mechanism of action involves pore formation in target bacterial membranes, leading to growth inhibition or lysis, while generally presenting a lower risk of resistance development compared with conventional antibiotics [136,137]. Also bacteriocins are mainly produced by lactic acid bacteria (LAB), a group of beneficial microbes commonly found in food and the human body, such as *Lactobacillus*, *Lactococcus*, *Pediococcus*, *Leuconostoc*, *Enterococcus*, *Streptococcus*, and *Bifidobacterium*, underlining their relevance within diet–microbiome–host interaction frameworks [138].

#### 3.5.2. Mechanisms of Action of Postbiotics and Probiotics

Postbiotics and probiotics exert notable immunomodulatory effects through multiple mechanisms engaging innate and adaptive immune responses. Most specifically, probiotics, as live microorganisms, interact with TLRs (Toll-like receptors), which are pivotal components of the host’s initial defense against microbial stimuli. For instance, recognition of probiotic ligands by TLR2, often through heterodimerization with TLR6, induces tolerogenic IL-10 secretion and anti-inflammatory signaling [139,140].

Key intracellular signaling cascades, including nuclear factor-kappa B (NF-κB) and mitogen-activated protein kinases (MAPKs) are affected by probiotics thereby facilitating communication with the host immune system. This modulation influences cytokine and chemokine profiles, contributing to the maintenance of immune homeostasis [141]. Similarly, postbiotics, particularly butyrate and propionate, exhibit substantial immunomodulatory effects. Butyrate enhances the diversity of regulatory T cells (Tregs), while propionate facilitates the expansion of peripheral Tregs within the intestinal mucosa. These effects contribute to epithelial homeostasis by modulating luminal pH, epithelial renewal, and local metabolic conditions too [119,142]. They also serve as key energy substrates for colonocytes and intestinal epithelial cells, supporting epithelial metabolism. Experimental evidence further suggests a role in regulating epithelial cell turnover, including pro-apoptotic effects in transformed cells, primarily demonstrated in preclinical models [143].

**Figure 5:**
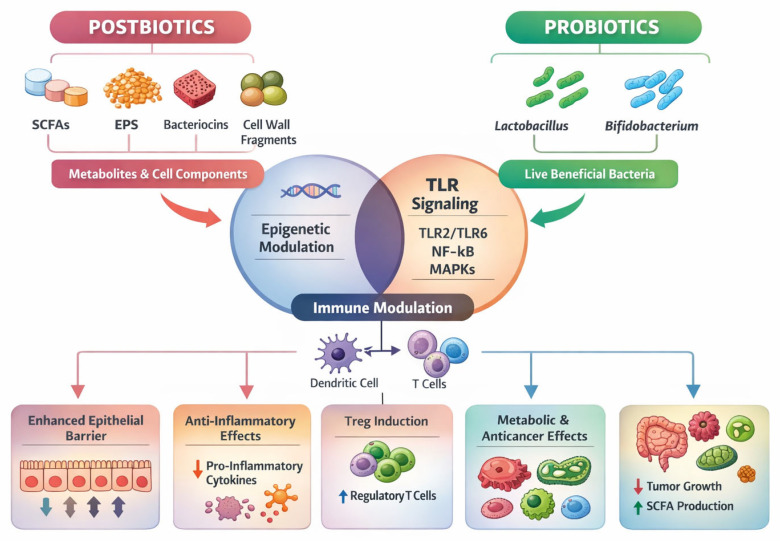
Mechanisms of action of postbiotics and probiotics on mucosal and systemic homeostasis. (This figure was created with Bio Render.com software, accessed date: 11 December 2025).

In Figure 5, we show the interconnected pathways through which postbiotics and probiotics influence mucosal and systemic physiology. Postbiotics, including short-chain fatty acids (SCFAs), exopolysaccharides (EPSs), bacteriocins, and bacterial structural components, exert their effects predominantly via metabolic signaling and epigenetic regulation, whereas probiotics act through live microbial interactions with host epithelial and immune cells, primarily via pattern-recognition receptors such as Toll-like receptors (TLRs). These upstream signals converge on key intracellular pathways, including NF-κB and MAPK signaling, leading to modulation of innate and adaptive immune responses, promotion of regulatory T-cell (Treg) activity, and attenuation of pro-inflammatory cytokine production. Downstream outcomes include reinforcement of epithelial barrier integrity, stabilization of microbial ecosystems, regulation of metabolic function, and protection against chronic inflammation. Collectively, the figure conceptualizes how diet–microbiome–host interactions mediated by postbiotics and probiotics support oral cellular homeostasis and broader systemic resilience.

### 3.6. Interventions Targeting Stress Biology, Oral Homeostasis, and Occupational Wellbeing

Interventions relevant to occupational wellbeing increasingly operate across two complementary levels: (i) modulation of stress physiology and mental health through scalable behavioral and digital strategies, and (ii) support of oral–systemic health through nutrition-focused approaches and structured coaching models that translate biological mechanisms into daily practice [99,100]. Digital lifestyle interventions show measurable benefits in stress, anxiety, depression, and broader wellbeing outcomes, supporting their role as scalable tools for prevention and early intervention in occupational contexts [100,101]. In parallel, non-pharmacological interventions used in healthcare environments, including mind–body and behavioral approaches, demonstrate clinically meaningful improvements in stress-related outcomes supporting a multi-component approach to workforce resilience [25,104,105]. Lifestyle programs targeting vulnerable occupational groups such as night-shift healthcare workers also indicate improvements in metabolic and mental health outcomes, consistent with evidence that circadian and behavioral optimization may reduce allostatic load [144].

From the oral–systemic health perspective, nutrition-based interventions and diet-oriented health models provide a coherent framework for linking dietary modulation, microbiome balance, and inflammatory control with oral health outcomes, which may indirectly support occupational resilience through reductions in chronic inflammatory burden [95,96,99,145]. Reviews focusing on functional foods, gut microbiome modulation, and metabolic health further emphasize that dietary strategies can shape host–microbe interactions relevant to inflammation and systemic regulation, particularly in populations characterized by metabolic vulnerability, such as individuals with diabetes [98]. Within dentistry, probiotic- and prebiotic-oriented approaches have been positioned not only as biological adjuncts but also as behaviorally mediated strategies that benefit from structured patient coaching, supporting sustained adherence and prevention-oriented oral health behaviors [97]. Coaching models developed for independent older adults illustrate that intervention effectiveness depends on implementation design, goal setting, tailored education, motivational support, and continuous feedback, rather than informational exposure alone, an approach that is transferable to occupational wellbeing programs in healthcare [95]. Finally, interventions targeting occupational wellbeing must also account for contextual and psychosocial determinants that shape stress trajectories, including caregiver burden and moral injury, which are increasingly recognized as high-impact determinants of occupational distress in healthcare and allied settings [102,103]. Ultimately, the evidence supports a layered intervention paradigm in which digital and behavioral stress-reduction tools, structured mind–body programs, circadian and lifestyle optimization, and nutrition- and coaching-based oral health strategies act synergistically to improve psychological outcomes while potentially influencing biological pathways linked to immune balance and systemic inflammation, although clinical validation of integrated oral-stress interventions remains limited [25,99,100].

### 3.7. Conceptual Funnel of Occupational Stress, Oral Cellular Homeostasis, and Professional Wellbeing

**Figure 6:**
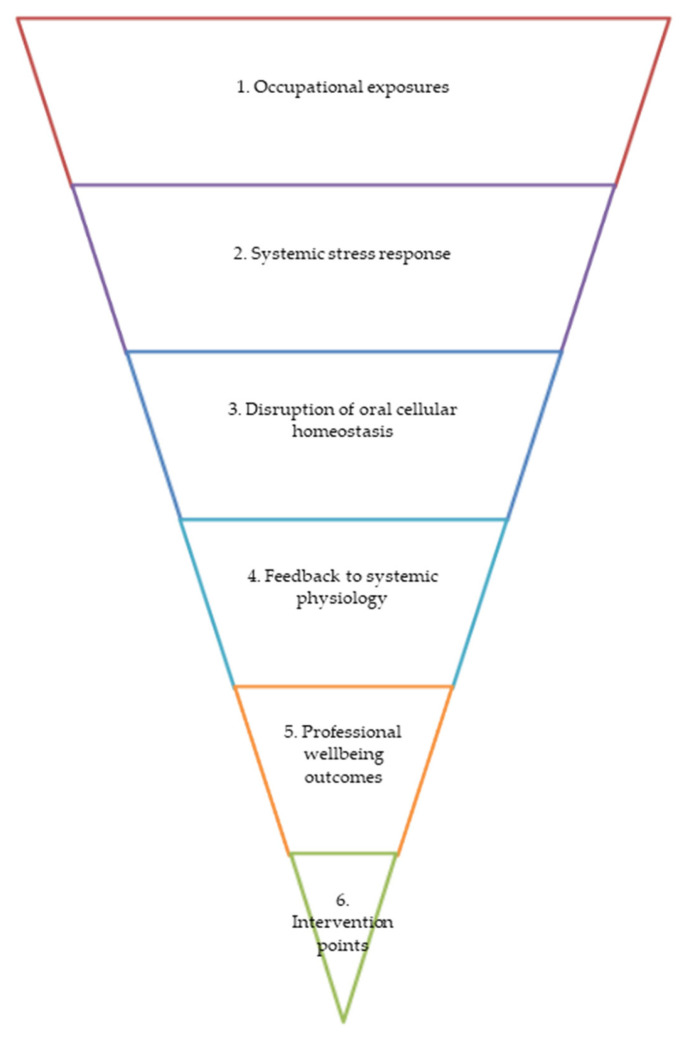
Conceptual pathway linking occupational stress to oral cellular homeostasis and professional wellbeing.

Figure 6 illustrates a sequential pathway whereby occupational exposures shape systemic stress physiology, disrupt oral cellular homeostasis (salivary, epithelial–immune, and microbiome mechanisms), and ultimately influence professional wellbeing. The current inverted-pyramid configuration highlights that intervention points appear last, occupying the narrowest section of the model, reflecting the empirical reality that most healthcare systems respond to stress after dysregulation has already occurred, that is, when burnout, fatigue, or biological strain becomes clinically visible [22,24]. The model is intentionally designed to allow conceptual reversal of the pyramid. When flipped, the intervention layer moves to the top, becoming the entry point rather than the final response. In this orientation, proactive measures, such as stress-modulating strategies, circadian alignment, microbiome-supportive behaviors, and saliva-based biomarker monitoring, act upstream to potentially stabilize systemic stress responses, support oral mucosal and microbial homeostasis, and reduce the risk of downstream wellbeing impairment [25,27,28,31]. Viewed in this reversible manner, the diagram emphasizes a central argument of this review: oral cellular mechanisms are not merely passive endpoints of occupational strain but early, sensitive interfaces where dysregulation may be detected at early stages and potentially controlled before professional wellbeing deteriorates. Conceptually shifting the model from a reactive to a preventive scaffold aligns with broader calls for integrative occupational health strategies that combine biological, psychological, and organizational approaches to strengthen the resilience of healthcare professionals, including dentists [85,86].

Figure 6 presents a conceptual and reversible pathway linking occupational stress to oral cellular homeostasis and professional wellbeing: 1. Occupational exposures (clinical workload, psychosocial stressors, time pressure, circadian disruption, infection control demands, and interpersonal demands in healthcare settings.). 2. Systemic stress response (activation of the HPA-axis with altered cortisol rhythms (including changes in the cortisol awakening response), sympathetic overactivation, low-grade systemic inflammation, and disturbed circadian alignment.). 3. Disruption of oral cellular homeostasis (a. salivary system, including changes in salivary cortisol, secretory IgA, cytokines (IL-6, TNF-α), antimicrobial peptides, and metabolic/proteomic signatures, as well as reduced buffering and antimicrobial capacity. b. Epithelial–immune barrier: Altered tight junction integrity, impaired mucosal repair, increased inflammatory signaling, and compromised innate immune responses. c. Oral microbiome: Reduced microbial diversity, dysbiosis, shifts toward pro-inflammatory or stress-responsive taxa, altered metabolite production (e.g., SCFAs, indole derivatives), and weakened ecological stability.). 4. Feedback to systemic physiology (amplification of systemic inflammation, altered neuroimmune communication, impaired stress recovery, microbiome–gut–brain axis signaling, and endocrine-mucosal crosstalk contributing to chronic allostatic load.). 5. Professional wellbeing outcomes (increased fatigue, burnout, emotional exhaustion, anxiety, depressive symptoms, cognitive overload, reduced resilience, reduced occupational engagement, and increased susceptibility to oral and systemic disease.).

### 3.8. Risk of Bias

The studies included in this review present methodological limitations that warrant cautious interpretation without diminishing their overall relevance. Much of the literature on oral microbiome dynamics, mucosal immunity, and occupational wellbeing is cross-sectional, limiting the ability to establish causal directionality between stress physiology, oral cellular mechanisms, and wellbeing outcomes [7,11]. Also, microbiome studies show substantial heterogeneity in sequencing methods and analytical pipelines, while salivary biomarker research often varies in sampling timing and diurnal control, contributing to between-study variability [28,30]. Furthermore, occupational wellbeing research frequently relies on validated self-report measures, which may introduce recall bias and subjective variability [24,85,86]. Although interventional studies employ more structured designs, modest sample sizes and short follow-up periods restrict conclusions regarding sustained physiological effects [26,27].

Accordingly, the strength of inference differs across domains. Associations between occupational stress and salivary cortisol or secretory IgA are consistent but largely observational, supported by established stress physiology rather than direct causal evidence. Findings involving inflammatory markers (e.g., IL-6, TNF-α) and oxidative stress similarly remain associative, with mechanistic support derived mainly from experimental models. Moreover, oral microbiome alterations are underpinned by strong ecological frameworks, yet human occupational studies are predominantly observational. In contrast, evidence on shift work and circadian disruption includes more interventional designs, strengthening causal inference for endocrine dysregulation, while downstream oral immune and microbiome effects remain less clearly established. Despite these limitations, convergence across diverse methodologies supports the conceptual relationships advanced in this review and highlights the need for longitudinal, multi-marker, and mechanistically integrated research to enhance causal resolution and translational relevance.

## 4. Discussion

This review integrates evidence across oral biology, occupational health, and behavioral science to support the concept that oral cellular homeostasis operates as a sensitive biological interface of occupational stress and resilience in healthcare professionals, with dentistry representing a high-exposure occupational model [25,85,86,145,146]. Across these domains, convergent findings indicate that chronic occupational strain is biologically embodied through HPA-axis activation, mucosal immune modulation, epithelial barrier alterations, and microbiome shifts, all of which are detectable in saliva and related oral biofluids [17,66,147,148]. Importantly, these biological signals exhibit dynamic temporal variability and may precede, or remain undetected by, self-report instruments, positioning saliva-based biomarkers as complementary, real-time indicators of physiological load in occupational settings [16,100,101].

### 4.1. Mechanistic Integration of Occupational Stress, Oral Cellular Homeostasis, and Resilience

As was revealed, biological plausibility is supported by a coherent mechanistic cascade in which occupational stress activates the HPA axis, elevating cortisol and sympathetic tone, with downstream suppression of mucosal immunity and disruption of epithelial integrity [30,34]. Reduced secretory IgA levels, altered cytokine profiles, and impaired tight-junction regulation compromise epithelial turnover and barrier function, thereby reshaping microbial ecology within the oral cavity [14,17,49]. Resultant dysbiosis and low-grade inflammation can, in turn, feed back into systemic stress pathways via neuroimmune and metabolic signaling, reinforcing a bidirectional amplification loop between stress biology and mucosal dysfunction [53,66,75,81]. While mechanistic plausibility supports a bidirectional stress-oral microbiome pathway, current human evidence remains largely associative, precluding definitive causal attribution between occupational stressors and microbial dysbiosis.

Within this framework, diet-derived modulators, particularly postbiotics such as short-chain fatty acids, exopolysaccharides, and bacteriocins emerge as biologically plausible modulators capable of stabilizing epithelial barriers, recalibrating immune signaling, and supporting microbial homeostasis [97,98,99]. Experimental and clinical nutrition studies further indicate that such microbial metabolites intersect immune, endocrine, and metabolic pathways, positioning nutritional strategies as modulators of both stress physiology and oral cellular resilience [45,61,74]. However, evidence supporting these strategies in occupational or clinical settings remains limited, and issues of feasibility, dosage, safety, and long-term effectiveness require validation in well-designed human studies.

Additionally, integration with psychological and resilience-oriented models strengthens the interpretation of these biological findings. From a resilience perspective, stable salivary IgA patterns, preserved epithelial integrity, and balanced microbiome profiles are associated with adaptive stress recovery and reduced susceptibility to burnout and emotional dysregulation [26,100]. This supports a biopsychosocial model in which biological integrity at the mucosal interface contributes to stress adaptation and emotional regulation, occupational sustainability, and mental well-being, rather than functioning solely as a passive marker of distress [27,101,103]. Causal interpretation of stress-related changes in salivary cytokines and inflammatory markers is limited by the predominance of cross-sectional human studies, although converging mechanistic and experimental evidence supports biological plausibility.

Key limitations of the current evidence base include the predominance of cross-sectional designs, modest sample sizes, heterogeneity in salivary sampling protocols and microbiome analytics, and a limited number of longitudinal or integrated multiomics studies [15,16,55]. Critically, most intervention studies do not employ oral biomarkers as primary outcomes, constraining causal inference and delaying translational implementation in occupational health frameworks [25,27]. These limitations report on the need for standardized saliva collection protocols (including timing and diurnal control), harmonized analytical pipelines, and integrative study designs that combine biological, psychological, and occupational metrics to advance precision prevention strategies in high-stress healthcare professions [13,99,144].

Implications for future research and occupational health practice are substantive and primarily research-oriented, emphasizing feasibility, monitoring, and prevention rather than therapeutic implementation. First, the deployment of salivary biomarker panels, including cortisol dynamics, secretory IgA, inflammatory cytokines, and targeted omics approaches, should be prioritized as early-warning indicators within occupational health surveillance frameworks, given their sensitivity to dynamic stress-related physiological changes [15,17,85,86,97,144,145,146,147]. Second, microbiome-informed monitoring should be incorporated for high-risk professional groups, as accumulating evidence links occupational strain with shifts in oral microbial ecology and mucosal immune balance [39,49,148,149,150,151,152].

Third, future interventions should adopt hybrid, systems-level designs that integrate stress-management strategies (e.g., mindfulness-based approaches, circadian alignment), targeted dietary and postbiotic interventions, and organizational or workload redesign, with outcomes evaluated against both biological (salivary, microbiome, immune) and psychological endpoints [26,99,144]. Finally, these findings support a shift towards a preventive, upstream occupational health model, moving beyond reactive burnout management to proactive stabilization of stress biology, mucosal immunity, and oral cellular homeostasis [22,27,96,97,98,99]. Collectively, this body of evidence repositions the oral cavity from a passive biomarker reservoir to an active biological interface within occupational stress physiology, offering feasible pathways for early detection, prevention, and resilience-building in healthcare workforces [13,14].

Overall, the evidence synthesized in this review supports consistent and biologically plausible associations between occupational stress and oral cellular homeostasis, while acknowledging that causal pathways remain to be confirmed through longitudinal and interventional research.

### 4.2. Operationalization of the Conceptual Framework

From a translational perspective, the proposed conceptual framework may be operationalized through the use of focused, non-invasive oral biomarker panels combined with standardized sampling protocols [28,30]. A core salivary panel for occupational stress monitoring in healthcare professionals may include salivary cortisol (diurnal profile or cortisol awakening response), secretory IgA, and selected inflammatory markers (e.g., IL-6, TNF-α), complemented where feasible by oral microbiome profiling or salivary metabolomic signatures [14,15,30,31,49,52]. Further, to enhance interpretability, saliva collection should account for key confounders, including circadian timing, recent food or caffeine intake, hydration status, smoking, acute oral inflammation, and routine oral hygiene practices, all of which are known to influence salivary biomarker variability [16,30]. Morning sampling within standardized time windows is generally preferred for endocrine markers, particularly cortisol, whereas repeated measures over time are required to capture dynamic stress-related changes rather than single-point estimates [30,31]. In occupational and healthcare settings, longitudinal rather than cross-sectional monitoring is particularly relevant. Periodic, low-burden saliva sampling aligned with work schedules (e.g., baseline, high-demand periods, and recovery phases) enables tracking of within-individual trajectories and stress recovery patterns, thereby reducing interindividual variability and strengthening interpretative validity [26,27,32]. Within this framework, the oral cavity functions as an accessible biological interface reflecting integrated endocrine, immune, and microbial responses to occupational strain [13,39,49]. Importantly, the model is intended to support hypothesis generation, early detection of dysregulated stress physiology, and evaluation of wellbeing-oriented interventions, rather than to serve as a diagnostic, therapeutic, or clinical decision-making tool [25,100]. Despite these integrative insights, several critical uncertainties remain.

### 4.3. Knowledge Gaps and Future Research Directions

Despite these integrative insights, several critical uncertainties remain. First, causal pathways linking occupational stress to oral cellular dysregulation cannot be established with confidence due to the predominance of cross-sectional designs. Second, the temporal ordering of endocrine, immune, and microbiome changes under chronic stress is largely unknown. Third, interindividual variability related to baseline oral health, diet, circadian disruption, and coping resources remains underexplored. Addressing these gaps will require longitudinal, multi-marker, and systems-level approaches.

## 5. Conclusions

This narrative review positions oral cellular homeostasis as a dynamic and sensitive biological interface linking occupational stress biology with immune, microbial, and epithelial regulation. The available evidence indicates that salivary biomarkers and oral microbiome patterns reflect physiological responses to occupational strain and may complement self-report measures by capturing stress-related biological variation in real time. Dietary and behavioral modulators, including postbiotics and stress-management interventions, emerge as biologically plausible strategies for supporting mucosal stability and overall wellbeing, although their clinical utility requires further validation. Integrating oral biomarker monitoring within preventive occupational health frameworks offers a promising conceptual approach for earlier identification of stress-related dysregulation and for informing targeted, resilience-oriented interventions in healthcare professionals.

## Figures and Tables

**Table 1:** Key articles grouped by thematic domains relevant to oral cellular homeostasis and occupational wellbeing.

Thematic Domain	Authors & Year	Study Type/Methods	Key Findings/Relevance
A. Oral Mucosal Immunity & Epithelial Barrier Biology			
Oral mucosal barrier mechanisms	[1] Lin et al., 2021	Mechanistic immunology review	Microbe–epithelial–immune crosstalk; foundation of oral homeostasis.
Physical & immune barriers of oral mucosa	[3] Şenel, 2021	Comprehensive immunity review	Defines physical, microbial, immune layers of the oral barrier.
Oral vs. gastrointestinal immune niches	[4] Suárez et al., 2021	Comparative immunology review	Highlights uniqueness of oral immune niche vs gut.
γδ T cells in oral diseases	[5] Wei et al., 2024	Immunology review	Shows γδ T cell roles in epithelial surveillance under stress.
Sensory–neuroimmune interactions	[6] Wang et al., 2024	Neuroimmune review	Demonstrates endocrine–neural modulation of mucosal immunity.
Oral innate immunity & epithelial cells	[34] Nittayananta, 2026	Immunology chapter	Maps epithelial innate mechanisms; core for homeostasis definition.
Oral mucosal barrier & systemic health	[39] Xin & Lei, 2026	Narrative review; mechanistic synthesis	Describes the oral mucosal barrier as a dynamic immune–epithelial interface regulating microbial balance, inflammation, and systemic signaling.
**B. Oral Microbiome Composition, Stability & Systemic Links**			
Lifespan mucosal–microbial interactions	[7] Iliev et al., 2025	Conceptual immunology review	Microbiota shapes mucosal immunity across lifespan.
Periodontitis microbiome biomarkers	[8] Radu et al., 2025	Narrative review	Links microbiome & salivary biomarkers to inflammation.
Hormonal effects on oral microbiome	[11] Rus et al., 2025	Clinical/experimental review	Shows endocrine modulation of oral microbiome; relates to stress.
Microbiome & wound healing	[11] Rus et al., 2025	Human biomarker study	Microbial shifts correlate with inflammatory wound healing responses.
Social capital-oral health-aging	[10] Liang & Gomaa, 2024	Cohort multi-omics	Oral health, cognition & aging interconnected biologically.
Tooth loss & cognitive decline	[9] Galindo-Moreno et al., 2022	Systematic review	Oral health linked to neurocognitive outcomes.
Gut–oral microbiome & periodontitis	[40] Yu et al., 2025	Meta-analysis of Mendelian randomization studies	Supports a causal role of gut microbiota composition in periodontitis risk, strengthening oral–systemic microbiome links.
Microbiome & viral-associated disease	[41] Zhang et al., 2025	Systematic review & meta-analysis	Demonstrates microbiome alterations in viral infection-associated inflammatory diseases, highlighting immune–microbial interactions.
Endodontic infections & microbiome	[42] Liu et al., 2025	Systematic review & meta-analysis (NGS-based)	Identifies Enterococcus as a persistent endodontic pathogen, emphasizing microbial resilience under inflammatory stress.
Prosthodontics & oral microbiome	[43] Madhan Kumar et al., 2025	Systematic review & meta-analysis	Shows dentures significantly alter oral microbiome composition and inflammatory profiles.
Immune signaling & barrier traversal	[44] Kulkarni et al., 2025	Meta-analysis of RNA-seq data	Identifies interferon-induced genes facilitating barrier crossing, relevant to mucosal immune vulnerability.
Nutritional modulation of immunity	[45] Lu et al., 2024	Randomized intervention study	L-glutamine supplementation enhances mucosal immunity and hormonal balance under physical stress.
Mucosal vaccination & immunity	[46] Flitter et al., 2025	Phase 2 placebo-controlled trial	Oral vaccination induces mucosal immunity and reduces viral shedding, validating oral immune responsiveness.
Oral mucosa & respiratory immunity	[47] Wang et al., 2024	Narrative review	Highlights oral microecological control of virus-related inflammatory responses.
Antiviral immunity in oral cavity	[48] Hickman & Moutsopoulos, 2025	Narrative review	Positions the oral cavity as an active antiviral immune site with systemic relevance.
Saliva–microbiome interactions	[49] Heller et al., 2025	Narrative review	Describes saliva as an active regulator of microbial stability and host defense.
Salivary metabolomics	[15] Zhao et al., 2025	Narrative review	Establishes saliva metabolomics as a non-invasive tool linking oral and systemic disease.
Microbial metabolites & immunity	[50] Schütz et al., 2025	Mechanistic review	Shows microbiome-derived metabolites modulate host immune responses and inflammation.
Oral epithelial innate immunity	[34] Nittayananta, 2026	Narrative review	Details epithelial cells as immune sentinels maintaining oral barrier homeostasis.
Saliva & systemic diagnostics	[13] Surdu et al., 2025	Narrative review	Supports saliva as a diagnostic medium for systemic and inflammatory diseases.
Oral immune responses	[14] Matsuoka et al., 2025	Experimental & narrative synthesis	Describes natural and induced immune mechanisms in saliva and oral tissues.
Micronutrients & emotional regulation	[51] Katta et al., 2024	Randomized controlled trial	Shows orally absorbed micronutrients reduce emotion dysregulation, linking nutrition and neurobiology.
Periodontitis & metabolomics	[52] Albahri et al., 2025	Observational metabolomics study	Links salivary metabolic profiles with chronic periodontal inflammation.
Saliva & cancer immunotherapy	[53] Nejat Dehkordi et al., 2025	Narrative review	Highlights salivary biomarkers as predictors of immunotherapy response.
Environment, stress & microbiome	[54] Shibata et al., 2025	Randomized controlled trial	Demonstrates environmental enrichment alters cortisol levels and microbiome composition.
Salivary transcriptomics	[55] Barnes et al., 2025	Methodological review	Positions saliva transcriptomics as a window into systemic gene regulation.
Saliva in precision dosing	[56] Xu et al., 2025	Methodological review	Validates saliva as a matrix for therapeutic drug monitoring.
Salivaomics in cancer	[57] Saravanan et al., 2025	Narrative review	Integrates proteomic, metabolomic, and transcriptomic saliva data for disease monitoring.
Salivary proteome & immunity	[18] Carneiro et al., 2026	Narrative review	Describes salivary proteins as mediators of immune defense and infection control.
Wound healing & microbiome	[58] Santamaria et al., 2025	Observational study	Links microbiome profiles and inflammatory biomarkers with oral wound healing outcomes.
Hormone diagnostics via saliva	[17] Ferrari et al., 2025	Narrative review	Establishes saliva as a reliable matrix for endocrine and stress hormone assessment.
Pediatric stress biomarkers	[59] Main et al., 2025	Narrative review	Identifies salivary markers as indicators of stress in dental settings.
Periodontal biomarkers	[60] Alavi et al., 2025	Narrative review	Positions salivary biomarkers as tools for early detection and precision dentistry.
Salivary diagnostics overview	[16] Albagieh et al., 2025	Narrative review	Summarizes applications, benefits, and limitations of salivary diagnostics.
**C. Integrated Evidence on Oral Microbiome, Mucosal Immunity, Saliva, and Systemic-Stress Biology**			
Nutritional modulation of microbiome & hormones	[61] Carter et al., 2025	Randomized controlled trial	Shows that specific dietary oligosaccharides concurrently reshape the microbiome, circulating hormones, and metabolic profiles, supporting diet-mediated regulation of immune–endocrine homeostasis.
Immune amplification therapies	[62] Steffin et al., 2025	Translational clinical study (CAR-T)	Illustrates how cytokine-enhanced immune signaling can reshape tumor-immune dynamics, highlighting systemic consequences of immune modulation relevant to mucosal immunity frameworks.
Anti-inflammatory dietary interventions	[63] Pardiñas López et al., 2025	Triple-blind randomized clinical trial	Provides clinical evidence that natural dietary agents exert antimicrobial and anti-inflammatory effects against periodontal pathogens, reinforcing nutrition as a modulator of oral inflammation.
Epithelial cytokine signaling	[64] McSorley & Hodge, 2025	Mechanistic parasitology review	Identifies epithelial-derived cytokines as central regulators of mucosal immune balance, relevant to stress- and infection-induced barrier modulation.
Antigen presentation & mucosal tolerance	[65] Hoelting et al., 2025	Mechanistic immunology review	Establishes antigen-presenting cells as key arbiters of tolerance versus inflammation at mucosal surfaces.
Metabolic syndrome & oral permeability	[66] Nehaoua et al., 2026	Narrative review	Links metabolic dysregulation with oral hyperpermeability and microbiome-driven inflammation, supporting oral–systemic disease integration.
Viral infection & mucosal immunity	[67] Shacklett, 2025	Narrative review	Highlights vulnerability and plasticity of mucosal immunity under acute viral stress.
Nanotechnology & immune modulation	[68] Jung & Son, 2025	Experimental/narrative review	Shows how nanoparticle-based strategies modulate mucosal immunity and microbiome interactions.
Anti-inflammatory cytokine regulation	[69] Branchett et al., 2024	Mechanistic immunology review	Positions IL-10 as a central regulator limiting mucosal inflammation across tissues.
Salivary cortisol & stress biology	[30] Dong et al., 2024	Systematic review	Defines methodological standards and limitations for using salivary cortisol as a stress biomarker.
Salivary exosomes	[70] Yu et al., 2024	Narrative review	Identifies salivary exosomes as emerging diagnostic and signaling mediators in oral disease.
Vaccine delivery via mucosa	[71] Laa et al., 2025	Narrative review	Describes protein nanocages as innovative platforms for mucosal immune activation.
T cell-epithelial crosstalk	[72] Bordoni & Fazio, 2025	Mechanistic genetics review	Explains how epithelial-T cell signaling regulates mucosal immune equilibrium.
Microbiota-dendritic cell interactions	[73] Letz et al., 2025	Mechanistic immunopathology review	Demonstrates microbiota-driven shaping of dendritic cell responses in chronic inflammation.
Flavonoids & oxidative stress	[74] Jomova et al., 2025	Mechanistic review	Links dietary flavonoids to redox balance and inflammatory control.
Polyphenols & inflammatory injury	[75] Wang L. et al., 2025	Experimental pharmacology study	Shows polyphenols mitigate inflammation and oxidative damage via cytokine modulation.
Oxidative stress & immune dysfunction	[76] Luo et al., 2025	Mechanistic immunology review	Establishes oxidative stress as a driver of immune-mediated tissue damage.
Vascular inflammation	[77] Shao et al., 2024	Mechanistic cell biology review	Demonstrates oxidative stress-induced microenvironmental disruption in chronic disease.
Inflammation & tumorigenesis	[78] Wang M. et al., 2025	Narrative review	Frames inflammation–oxidative stress axis as a universal oncogenic driver.
Oxidative stress in cancer	[79] Tomaziu-Todosia Anton et al., 2025	Narrative review	Reviews antioxidant strategies targeting inflammatory carcinogenesis.
Infection, inflammation & fertility	[80] Potiris et al., 2025	Narrative review	Highlights systemic consequences of chronic inflammation and oxidative stress.
Inflammation & mental health	[81] Teixeira et al., 2025	Narrative review	Positions inflammation as a biological substrate of late-life depression.
Anxiety & aging	[82] Johnco et al., 2024	Clinical psychiatry review	Connects stress-related disorders with biological aging processes.
Oxidative stress biomarkers	[83] Aravapally et al., 2025	Methodological review	Discusses advanced strategies for quantifying oxidative stress in complex systems.
Gingival immune regulation	[84] Hsu et al., 2025	Narrative review	Describes immune and tissue dynamics in experimental gingivitis.
**D. Occupational Stress, Wellbeing & Biomarkers**			
Stress, anxiety, depression, resilience & spiritual wellbeing in students	[85] Mangoulia et al., 2025	Cross-sectional observational study	Reports high psychological burden among dental & nursing students post-pandemic; highlights resilience & hope as protective factors; relevant to occupational wellbeing trajectories.
Quality of life & wellbeing in academic dental personnel	[86] Antoniadou, Mangoulia & Myrianthefs, 2023	Cross-sectional study comparing academic staff & service quality	Demonstrates associations between professional wellbeing, QoL parameters, and perceived quality of academic services; supports link between occupational strain and performance outcomes.
Circadian dysregulation & burnout	[22] Ungurianu & Marina, 2025	Systematic review	Burnout affects circadian biology; linked to cortisol.
Immune & mucosal function under stress	[23] Zhang et al., 2025	Immunology review	Chronic stress disrupts systemic and mucosal immunity.
Burnout, depression & suicide in HCWs	[24] Nguyen et al., 2025	Scoping review	High burden of psychological distress in healthcare.
Stress-reduction therapies	[25] Meneses Damasceno et al., 2025	Integrative review	Interventions improve physiological stress markers.
Tele-yoga for HCWs	[26] Naveen et al., 2024	Randomized controlled trial	Improves cortisol and immune markers.
Night-shift worker intervention	[27] Robinson et al., 2025	Randomized crossover trial	Improves salivary cortisol and metabolic responses.
Salivary biomarkers in occupational medicine	[28] Koh & Koh, 2007	Landmark review	Classic reference establishing saliva as biomarker matrix.
Salivary cortisol methodology	[30] Dong et al., 2024	Systematic methodological review	Timing, diurnal variation, sampling, assay accuracy.
Cortisol awakening response	[31] Stalder et al., 2025	Endocrine review	CAR as reliable HPA-axis stress indicator.
Environmental richness & cortisol	[54] Shibata et al., 2025	Randomized trial	Environment modulates cortisol & microbiota.
Salivary biomarkers & occupational fatigue	[87] Mahdavi et al., 2025	Observational study; salivary biomarker assessment	Demonstrates associations between salivary biomarkers and occupational fatigue, supporting saliva as a non-invasive indicator of work-related physiological strain.
**E. Dietary, Nutritional, and Food-Related Modulators of Oral Cellular Homeostasis**			
Diet–oral microbiota interaction	[88] García HC et al., 2025	Systematic review	Diet is a key determinant of oral microbiota composition; fiber-rich diets support microbial diversity and immune balance, whereas high-sugar patterns promote dysbiosis and inflammation.
Nutrition and oral–periodontal health	[89] Curca FR et al., 2026	Narrative review	Links nutrition to oral and periodontal health through immune modulation, oxidative stress control, and microbiome-driven regulation of epithelial integrity and inflammation.
Dietary inflammatory potential and microbiome	[90] Liu J et al., 2025	Observational study with microbiome profiling	Higher dietary inflammatory potential is associated with altered oral and gut microbiota and increased systemic inflammation.
SCFAs and microbiome–host crosstalk	[91] Nireeksha et al., 2025	Narrative mechanistic review	Identifies SCFAs as metabolic mediators linking diet to immune regulation, epithelial energy metabolism, and microbial stability.
Oral–gut microbiome axis	[92] Parveen S et al., 2024	Narrative review	Examines oral-gut microbiome crosstalk and the role of diet in shaping microbial ecology and immune signaling.
Dietary patterns and periodontitis	[93] Mao J.-S. et al., 2025	Narrative review	High-fiber, anti-inflammatory diets protect against periodontal inflammation via SCFA production and immune modulation.
Diet and oral microbiome	[94] Santonocito S et al., 2025	Book chapter/narrative synthesis	Dietary composition shapes oral microbiome structure, inflammatory signaling, and disease susceptibility.
**F. Interventions Targeting Stress Biology, Oral Homeostasis, and Occupational Wellbeing**			
Oral health coaching & dietary interventions	[95] Antoniadou & Varzakas, 2020	Narrative review; coaching models	Structured oral health and diet coaching improves adherence, self-management, and preventive oral health behaviors in independent elderly populations.
Diet, oral health, and systemic links	[96] Antoniadou & Varzakas, 2021	Narrative review	Highlights diet as a modulator of oral and systemic inflammation, microbiome balance, and chronic disease risk.
Probiotics, prebiotics, and caries prevention	[97] Amargianitakis et al., 2021	Narrative review	Integrates probiotic-based strategies with patient coaching to support caries prevention and oral microbial balance.
Functional foods, gut microbiome, and metabolic health	[98] Bezirtzoglou et al., 2021	Systematic review	Demonstrates the role of microbiome-targeted functional foods in metabolic regulation and inflammatory control, with implications for oral–systemic health.
Dietary interventions for disease prevention	[99] Antoniadou & Varzakas, 2024	Narrative review	Presents dietary strategies as tools for reducing systemic and oral disease burden via immune and microbiome modulation.
Digital lifestyle interventions & mental health	[100] Brinsley et al., 2025	Systematic review & meta-analysis	Digital interventions significantly reduce stress, anxiety, and depression, supporting scalable occupational wellbeing strategies.
Digital interventions beyond depression	[101] Linardon et al., 2025	Meta-analysis	Shows broader wellbeing benefits of digital mental health interventions beyond depressive symptom reduction.
Caregiver burden and psychosocial stress	[102] Gholami et al., 2025	Scoping review	Identifies unmet psychosocial and emotional needs contributing to chronic stress and burnout in caregiving roles.
Moral injury prevention	[103] Williamson et al., 2025	Narrative review	Frames moral injury as a distinct stressor requiring targeted preventive and therapeutic interventions.
Mind–body interventions & immune markers	[26] Naveen et al., 2024	Pilot randomized controlled trial	Tele-yoga reduced burnout and improved immune markers in healthcare workers, linking stress reduction with biological outcomes.
Non-pharmacological stress interventions	[25] Meneses Damasceno & Pimentel, 2025	Integrative review	Summarizes effectiveness of non-pharmacological stress-reduction strategies in healthcare workers.
Breathwork and stress resilience	[104] Little, 2025	Narrative review	Breath-based interventions enhance stress resilience and emotional regulation through autonomic modulation.
Lifestyle, circadian disruption, and metabolic health	[27] Robinson et al., 2025	Randomized crossover trial	Lifestyle intervention improved metabolic and mental health outcomes in female night-shift healthcare workers.

## Data Availability

No new data were created or analyzed in this study.

## Supplementary Materials

The following supporting information can be downloaded at: https://www.mdpi.com/article/10.3390/cells15050406/s1, Table S1: Full electronic search strategy.
