# Supplementary material for: Oral Cellular Homeostasis and Occupational Wellbeing in Healthcare Professionals Under the Lens of Salivary, Immune, and Microbiome Mechanisms
Source: Cells. 2026 Feb 26;15(5):406. doi: 10.3390/cells15050406 (PMC12984415; doi:10.3390/cells15050406)
Supplement: Supplementary file 1 [file cells-15-00406-s001.zip › cells-4126511-supplementary.pdf]

Table S1. Full electronic search strategy

Database	Search string (exact)	Date limits	Language	Final search date
**PubMed/MEDLINE**	(“oral mucosa” OR “oral immunity” OR saliva* OR “salivary biomarkers” OR microbiome OR “oral microbiome”) AND (“occupational stress” OR burnout OR fatigue OR wellbeing OR resilience) AND (“healthcare professionals” OR dentists OR dental professionals OR nurses OR physicians OR clinicians)	January 2020-January 2026	English	31 January 2026
**Scopus**	TITLE-ABS-KEY(oral AND (saliva* OR “salivary biomarkers” OR “oral immunity” OR microbiome) AND (“occupational stress” OR burnout OR wellbeing OR fatigue) AND (“healthcare professionals” OR dentists OR nurses OR physicians))	2020-2026	English	31 January 2026
**Web of Science Core Collection**	TS=(oral AND (saliva* OR “salivary biomarkers” OR microbiome OR “oral immunity”) AND (“occupational stress” OR burnout OR fatigue OR wellbeing) AND (“healthcare professionals” OR dentists OR nurses OR physicians))	2020-2026	English	31 January 2026
**Cochrane Oral Health**	oral AND (saliva OR “salivary biomarkers” OR microbiome) AND stress	Inception - January 2026	English	31 January 2026
